# Melatonin effect during different maturation stages of oocyte and subsequent embryo development in mice

**Published:** 2013-01

**Authors:** Mohammad Hadi Bahadori, Fatemeh Ghasemian, Mina Ramezani, Zakieh Asgari

**Affiliations:** 1 *Cellular and Molecular Research Center, Faculty of Medicine, Guilan University of Medical Sciences, Rasht, Iran.*; 2 *Department of Biology, Biology Faculty, Kharazmi (Tarbiat Moallem) University, Tehran, Iran.*; 3 *Department of Biology, Faculty of Sciences, Ashtian Branch, Islamic Azad University, Ashtian, Iran.*; 4 *Department of Anatomy, Faculty of Medicine, Guilan University of Medical Sciences, Rasht, Iran.*

**Keywords:** *In vitro oocyte maturation*, *Development*, *Invitro fertilization*, *Melatonin*, *Cumulus-oocyte complex*

## Abstract

**Background: **It is important to protect oocytes and embryos from oxidative stress in the culture medium. Melatonin has been shown to be a direct free radical scavenger.

**Objective:** Effect of melatonin during in vitro oocyte maturation, fertilization and embryo development of mouse oocytes was evaluated.

**Materials and Methods:** Oocytes from supper-ovulated mouse were divided to two groups: cumulus oocyte complexes (COCs, group I) and denuded COC (d-COCs, group II). The oocytes were cultured in maturation medium with different doses of melatonin (1×10^1^-10^5^ nM). The cumulus expansion and nuclear status were evaluated after 24 h of in-vitro maturation. The oocytes were used for in-vitro fertilization. The fertilized oocytes were cultured in medium supplemented with different doses of melatonin.

**Results:** The expansion (86.79%) and maturation (80.55%) rate of COCs increased in supplemented medium with 10 nM of melatonin vs. control group (73.33%), p=0.006 and p=0.026 respectively), but oocytes without cumulus cells indicated higher maturation rate at higher melatonin doses (10 and 100 M, 84.34% and 79.5% respectively( vs. 69.33% in control group (p=0.002). Fertilization rate was higher in treated medium with 1 μM of melatonin (93.75%, p=0.007). The rate of cleavage and blastocyst formation was promoted in medium supplemented with 10 and 100 nM of melatonin (92.37% and 89.36% vs. 81.25% in control group, p=0.002). We observed a dose dependent response to melatonin treatment in this experiment.

**Conclusion:** Exogenous melatonin can promote cumulus cell expansion, in vitro oocyte maturation, and embryo development. However we investigated a dose-dependent response in different stages of maturation and development. It may reflect sensitive rate of oocytes and embryos to culture conditions.

## Introduction

Events such as exposure to light, elevated oxygen concentrations, and unusual concentrations of metabolites and substrates can cause oxidation stress in oocytes and embryos following of culture conditions. Reactive oxygen species (ROS) damage cell membranes and DNA and can cause apoptosis (1). 

Specially, free radicals function affect microenvironments of oocytes, and sperm. Therefore, these microenvironment changes in the follicular fluid (FF) influence on follicular development, ovulation, quality of oocytes, sperm oocyte interaction, implantation, and early embryonic development (2). It is important to protect oocytes and embryos from oxidative stress in the culture medium and for this purpose, antioxidant compounds are a candidate approach. 

The presence of an antioxidant or radical scavenger in in-vitro culture medium could decrease oxidative stress and promote culture conditions. Melatonin (N-acetyl-5-methoxytryptamine), which was discovered about 50 years ago, is an endogenous compound synthesized by the pineal gland in the human brain. Melatonin has been shown to be a direct free radical scavenger and indirect antioxidant via its stimulatory actions on antioxidant system (3-5). 

Ishizuka *et al* reported positive influence of melatonin on mouse embryo development in-vitro and found that melatonin at the concentrations from 10^-6^-10^-8^ M supports fertilization and early in vitro development of mouse embryos. It has also been reported that melatonin plays beneficial effects on nuclear and cytoplasmic maturation during porcine in-vitro oocyte maturation (IVM). Melatonin is present in human preovulatory follicular fluid at the concentrations of 3-fold higher than in peripheral serum (6). 

It has been reported that melatonin is soluble both in water and in lipids and hence, acts as a hydrophilic and hydrophobic antioxidant. Therefore, in this study culture media were placed in well dishes to keep melatonin effects during culture. In this study, also the effect of different melatonin concentrations on both maturation and development rate for same oocytes has been investigated. Therefore, the specific objective of this study was to evaluate effects of melatonin supplementation on oocytes with/without cumulus cells during mouse IVM, especially on cumulus cells expansion and nuclear maturation in commercial tissue culture medium 199. 

Then, the fertilization rate of the matured oocytes has been analyzed. At the end, the development rate of resulted embryos was studied in medium supplemented with different doses of melatonin. This study is the first study that evaluates the melatonin effect from maturation until developmental stage considering the presence or absent of cumulus cells in mice oocytes, IVM.

## Materials and methods

**Collection of COCs and d-COCs**

This study was an original research as experimental intervention. Fifty mice were kept with a 12h light/dark cycle. They were kept at a constant temperature with unrestricted access to food and water. All animals were treated in accordance with the guidelines of the Guilan University of Medical Science (GUMS), University Ethics Community Standards on the Care and Use of Laboratory Animals. Female NMRI (Naval Medical Research Institute) mice, 6-8 weeks old, were superovulated by intraperitoneal injection of 5 IU of pregnant mares’ serum gonadotrophin (PMSG; Organon, Holand). 

After 48 hours, ovaries were removed and immediately placed in pre-warmed tissue culture medium-199 (TCM-199 plus NaHCO3, penicillin and streptomycin, Sigma) supplemented with 5% fetal calf serum (FCS, Sigma). Oocytes were mechanically isolated out of ovaries from mice and were divided into two groups: I) COCs (cumulus oocyte complexes), and II) d-COCs (oocytes without cumulus cells). The follicles were washed in the culture medium before being randomly plated in individual wells. 

**In vitro maturation**

In group I, only oocytes enclosed in a compact cumulus with evenly granulated cytoplasm for maturation were selected. The COCs and d-COC were washed 3 times in oocyte collection medium and one time in oocyte maturation medium (bicarbonate-buffered TCM199) supplemented with 5% FCS. Oocyte maturation was performed by culturing approximately 10-15 oocytes in maturation medium in wells at 37^o^C in 5% CO_2_ and 95% humidity for 24 h. 

Melatonin stock solution was prepared with an ethanol/TCM199 system. 23.23 mg melatonin (Sigma) was first dissolved in 1.0 ml absolute ethanol and diluted in TCM199 by serial concentration. In this way 10 nM, 100 nM, 1 μM, 10 μM and 100 μM melatonin stock solution was prepared. The stock solutions were stored in refrigerator at 4^o^C for no longer than 2 weeks. The amount of ethanol in our experiment was 0.1% in the maturation medium and we used it as vehicle group.

**Assessment of the cumulus expansion and nuclear status**

COCs were evaluated in order to assay cumulus expansion by phase contrast invert microscope (Olympus, Japan). Some of the COCs showed fully expanded cumulus cells after 24 h maturation period. These COCs were assessed and those which COCs were not expanded or showed incomplete expansion did not account. Then oocytes were used for fertilization. 

**Figure 1:**
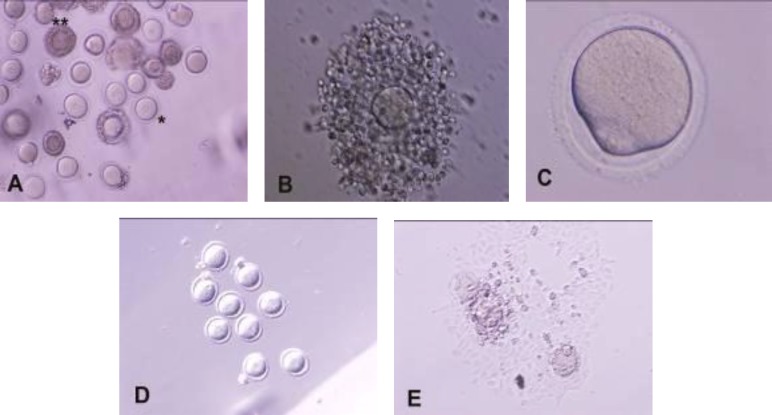
Mouse oocytes at different maturation stages. (A) oocytes with and without cumulus cells in germinal vesicle stage (COCs: double star and d-COC: star), (B) expanded COC, (C) metaphase I, (D) GVBD and (E) MII (A,D,E:  100 and B,C:  200

To assay nuclear status, oocytes without cumulus cells first assessed for perivitellin space and first polar body excursion by phase contrast invert microscope. The oocytes withpolar body account as matured and metaphase-II oocytes. Oocytes without polar body were recovered. Oocytes were classified as germinal vesicle (GV, with a nucleus), germinal vesicle breakdown (GVBD, without nucleus and polar body), metaphase I (MI, without nucleus and polar body and with blocked first polar body), and metaphase II (MII, with first polar body) stages of the maturation process (Figure 1). After the evaluations, oocytes were immediately used for in-vitro fertilization (IVF).

**Experimental design**

In this study different concentrations of melatonin (0, 10 and 100 nM, 1, 10 and 100 μM) and 0.1% ethanol (vehicle) were added in the maturation and development media. 

**In vitro fertilization**

After 18 h of IVM, COCs and d-COC were washed twice in fertilization media [T6 Media and 15mg/ml bovine serum albumin (BSA), Sigma] and co-incubated with capacitated sperms from mice in treated groups with different doses of melatonin (as mentioned above). Sperms were obtained from adult male NMRI mice by epididymal extraction, followed by incubation of the in vitro matured oocytes with these sperms adjusted to one million sperms/ml (4-5 oocytes per fertilization drop) and then inseminated into the central dish of T6 medium containing 15mg/ml of BSA.

**Embryos development**

After 4-6 h of insemination, the inseminated oocytes were removed from insemination medium and cultured in groups of 5-10 zygotes per wells of T6 medium containing 4mg/ml of BSA and different doses of melatonin (0, 10 and 100 nM, 1, 10 and 100 μM) at 37^o^C and 5% CO_2_. Fertilization rates were scored 20 h after IVF and 2-cell embryos transferred to fresh medium for another 25-27 h, after which embryos were assessed for their rate of development, to distinguish percentage of faster developing embryos and finally moved into fresh medium for 47-49 h. The embryos were counted at the end of the culture period at 96-100 h post fertilization in order to determine blastocyst development (7). In all stages, the development medium was supplemented with different doses of melatonin and all the experiments were repeated seven times. 

**Statistical analysis**

All outcomes were assessed using Chi-square test. All statistical analyses were performed using the Statistical Package for the Social Sciences (SPSS, version 13.0 for windows). Differences were analyzed in COCs expansion, oocyte maturation, and cleavage and blastocyst rates with a significance level of 0.05. 

## Results

**Expansion of cumulus and perivitellin space**

In group I after culturing in six different doses of melatonin containing media (0, 10, 100 nM, 1, 10, 100 M) for 24 h, over 86.79% of COCs had full cumulus cell expansion in maturation medium supplemented with 10 nM of melatonin. There is significantly difference between treated groups with 10 nM of melatonin and control group (p=0.006). By increasing melatonin concentration, the degree of cumulus expansion decreased (Table I).

In group II, matured oocytes in control and other groups were clearly visible with presence of first polar body in perivitellin space (they have been considered as the metaphase-II stage oocytes). 

**Evaluation of meiotic maturation**

Progress and maturation percentage of oocytes are indicated in table II. Maturation rate of COCs incubated in 10 nM of melatonin-containing media for 24 h resulted in 80.55%, which was different from the control (73.33%, p=0.026). When melatonin concentrations were increased to 10 and 100 μM, maturation rate of COCs significantly decreased to 46.36% and 50% (p=0.07 as compared to the control group). Regarding the results of supplemented groups with 10 and 100 μM, maturation rate may decrease, when melatonin concentrations were increased higher than 100 μM compared to control group. Maturation rate of oocytes without cumulus cells cultured in supplemented medium with 10 and 100 μM reached to 84.34% and 79.5% respectively as compared to control group (69.33%, p=0.002). 

There was significant difference among groups at GVBD and metaphase-I stages in treated groups with 10 and 100 μM, as compared to control group (p=0.004). Therefore, higher concentrations of melatonin can promote maturation rate in oocytes without cumulus. A dose dependent response to melatonin treatment in this experiment has been seen. Absolute ethanol was used for diluting melatonin (0.1% as vehicle group), that had no detrimental effect on neither cumulus cell expansion nor nuclear status (p=0.08).

**Evaluation of fertilization and development**

Two cell embryos indicated the rate of successful fertilization from IVF. The progressive developmental percentage of embryos has been shown in table III. The successful fertilization rate was higher in treated group with 1 µM of melatonin (93.75%, p=0.003) in comparison to control group. The morula development rate from two-cell embryos increased in supplemented medium with 10 and 100 nM (94.06% and 91.48% respectively) as compared to control group (85.43%, p=0.034). The percentage of the embryos reached to blastocyst stage were higher in 10 and 100 nM treated groups (92.37% and 89.36% respectively) than the control group (81.55%, p=0.002).

**Table I:** Effect of melatonin on cumulus expansion of mouse COCs, in vitro

**Melatonin concentrations**	**No. of COCs**	**Cumulus expansion [n (%)]**	**M-II [n (%)]**
0 (control)	105	84 ± 10.8 (80)	77 ± 9.4 (73.33)
0 (vehicle)	102	82 ± 9.8 (80.39)	76 ± 8.9 (74.48)
10 nM	180	156 ± 16.4 (86.66)^a^	145 ± 16.9 (80.55)^b^
100 nM	170	144 ± 14.3 (84.7)	130 ± 14.6 (76.47)
1 M	105	88 ± 9.2 (83.8)	70 ± 8.1 (66.66)
10 M	110	90 ± 9.7 (81.81)	51 ± 6.1 (46.36)
100 M	110	89 ± 9.4 (80.90)	55 ± 6.9 (50)

COCs: cumulus-oocyte complex, M-II: metaphase-II.

a, b within the same column, values with different letters were significantly different in comparison to control group. ^a^) p < 0.006 and ^b^) p < 0.026.

**Table II:** The maturation rate of d-COC oocytes in melatonin containing-media

**Melatonin** **concentrations**	**No. of d-COC**	**Nuclear statues after 24 h (Mean±SE)**			
		**GV [n (%)]**	**GVBD [n (%)]**	**M-I [n (%)]**	**M-II [n (%)]**
0 (control)	75	0	58 ± 6.2 (77.33)	56 ± 6.4 (74.66)	52 ± 5.9 (69.33)
0 (vehicle)	98	0	76 ± 8.4 (77.55)	75 ± 7.9 (76.53)	70 ± 6.9 (71.42)
10 nM	111	0	90 ± 9.7 (81.08)	87 ± 8.9 (78.37)	82 ± 7.9 (73.87)
100 nM	90	0	74 ± 7.6 (82.22)	67 ± 6.7 (74.44)	67 ± 6.9 (74.44)
1 M	90	0	71 ± 8.4 (78.88)	58 ± 6.5 (64.44)	58 ± 6.1 (64.44)
10 M	115	0	104 ± 14.8 (90.43)^a^	97 ± 10.2 (84.34)^a^	97 ± 10.9 (84.34)^a^
100 M	122	0	106 ± 13.9 (86.88)^a^	97 ± 11 (79.5)^a^	97 ± 10.4 (79.5)^a^

GV: germinal vesicle, GVBD: germinal vesicle break down, M-I: metaphase-I, M-II: metaphase-II.

a within the same column, values with different letters were significantly different in comparison to control group. ^a^) p < 0.004.

Chi-square test.

**Table III:** Effect of melatonin on IVF and development rate of mouse embryos, in-vitro

**Melatonin concentrations**	**No. of two-cell (%)**	**No. of morula (%)**	**No. of blastocyst (%)**
0 (control)	103 (80.62)	88 ± 8.7 (85.43)	84 ± 7.5 (81.55)
0 (vehicle)	113 (77.93)	104 ± 12.3 (84.55)	102 ± 11 (82.92)
10 nM	118 (51.98)	111 ± 12.5 (94)^b^	109 ± 11.7 (92.37)^c^
100 nM	141 (71.57)	129 ± 14.6 (91.4)^b^	126 ± 14.3 (89.36)^c^
1 M	120 (93.75)^a^	104 ± 11.6 (86.66)	99 ± 9.4 (82.5)
10 M	100 (67.56)	87 ± 7.6 (87)	82 ± 6.2 (82)
100 M	100 (65.78)	86 ± 7.8 (86)	84 ± 6.9 (84)

a, b within the same column, values with different letters were significantly different in comparison to control group.

^a^ p<0.003, ^b^ p<0.034 and ^c^ p<0.002.

Chi-square test.

## Discussion

On the basis of the evidence in different studies, melatonin is potentially useful in culture condition as direct scavenging of free radicals (2). Melatonin has the effect of: inhibition of the activity of a pro-oxidative enzyme, stimulation of the activity of antioxidant enzymes, distribution in all tissue, cells and cellular compartments throughout the organism, and rapid diffusion through all biological membranes (8-10). The results of this study indicate that COCs need lower concentration of melatonin (10 nM) during maturation stages in comparison to oocytes without cumulus cells. 

These oocytes without cumulus cells indicated higher maturation rate in treated maturation medium with 10 and 100 µM. The resulted maturation rate of COCs may reflect the positive role of cumulus cells, too. In the other word, in the presence of cumulus cells, COCs can progress even in low melatonin dose. The maturation rate of oocytes without cumulus cells promote in doses 10 and 100 µM during GVBD, M-I and M-II stages. This result is in agreement to results of Kang *et al* (2009), that indicated the antioxidant effects of melatonin supplementation (10 ng/ml) during IVM of porcine COCs which resulted in a greater proportion of oocytes extruding the polar body in comparison to control group (75.6% vs. 84.6%) and beneficial effects of melatonin were shown on nuclear and cytoplasmic maturation. 

However, it appears the treatment cannot influence on cleavage frequency and blastocyst cell number. Kang *et al* also indicated bovine cumulus and granulosa cells express melatonin receptor 1 (MT1) gene (11). In other study, Takada *et al* indicated that melatonin during IVM protects the bovine cumulus cells from DNA damage but this effect did not influence embryo development, in vitro (12). Also the effect of melatonin was assayed on different development stages. 

Our results demonstrate that the embryos need lower melatonin concentrations. These results may express that embryos can resist elevated oxygen concentrations and unusual concentrations of metabolites and substrates in culture conditions. 

Therefore, embryo culture medium need lower melatonin concentrations. Better development of embryos in lower melatonin concentration observed in blastocyst stage. To our knowledge, the present study is the first report on the use of melatonin in the culture of mouse oocytes to embryos continuously. Also, this study assayed the effect of melatonin on oocytes with/without cumulus cells maturation rate. 

Melatonin is a documented powerful free radical scavenger and a broad-spectrum antioxidant (13, 14). The ovarian functions may directly influence by melatonin, so that the melatonin concentration in human ovarian follicular fluid (FF) is higher than plasma, and the steroidogenesis activity of granulose cells (GCs) and follicular function were altered in hen, hamster, and humans by melatonin (15-18). 

The ovary possibly synthesized melatonin and released into FF. However, it appears that the melatonin presence in the ovary and preovulatory FF is derived from the circulation (2). One problem in female infertility is poor quality of oocytes. The follicles, especially during the ovulatory process produce ROS (19). Therefore, the cell membrane lipids and DNA are destroyed by ROS, progresses apoptosis quickly, occurs two-cell block, and finally inhibits fertilization (20, 21). The presence of high melatonin levels in FF and the presence of melatonin receptors in GCs suggest that this indoleamine may be a molecule that is highly beneficial in the follicle (18, 22). 

Melatonin also regulates the oocyte maturation capacity (23). Under the influence of maturation-promoting factor, the breakdown of the oocyte nuclear envelope or germinal vesicle breakdown (GVBD) during prophase to metaphase stages and the meiotic cell cycle progression of oocytes were considered as oocyte maturation progression and demonstrated as morphological changes (24). Melatonin (50-500 pg/mL) accelerated the action of maturation-inducing hormone on the maturation-promoting factor and GVBD of oocytes (25). Melatonin in the culture medium supports not only mouse fertilization but early development of embryonic tissue (6). The dosage of 10 nM melatonin is effective on porcine embryo cleavage rates and blastocyst total cell numbers positively. 

On the other hand, the other concentration of melatonin (1 pM-100 µM) do not show negative effects on embryo development or even during pregnancy in presence of higher doses (26, 27). Ishisuka *et al* reported that development rate of embryos increase during two cells to blastocyst stages when the mouse embryos were cultured in a medium containing 10^-8^ or 10^-6^ M melatonin (6). Adriaens *et al* indicated that a dose of 10 µM could be suitable to reduce oxidative stress in cultured follicles (23). On the basis of our results, the presence of melatonin in culture medium could also promote maturation of oocytes and development of mouse embryos, too.

## Conclusion

The results of this study show that the administration of exogenous melatonin during IVM, IVF and embryonic stages improves the oocyte maturation and embryo development. It is also concluded that melatonin increases cumulus cell expansion and maturation, embryo cleavage and blastocyst formation rates. However we investigated a dose-dependent response in different stages of maturation, fertilization and development. It may reflect sensitive rate of oocytes and embryos to culture conditions. 
